# The endoplasmic reticulum: structure, function and response to cellular signaling

**DOI:** 10.1007/s00018-015-2052-6

**Published:** 2015-10-03

**Authors:** Dianne S. Schwarz, Michael D. Blower

**Affiliations:** Department of Molecular Biology, Massachusetts General Hospital, Boston, MA 02114 USA; Department of Genetics, Harvard Medical School, Boston, MA 02115 USA; New England Biolabs, Ipswich, MA 01938 USA

**Keywords:** Interphase, Mitosis, Unfolded protein response, Organization, Fertilization, Phosphorylation

## Abstract

The endoplasmic reticulum (ER) is a large, dynamic structure that serves many roles in the cell including calcium storage, protein synthesis and lipid metabolism. The diverse functions of the ER are performed by distinct domains; consisting of tubules, sheets and the nuclear envelope. Several proteins that contribute to the overall architecture and dynamics of the ER have been identified, but many questions remain as to how the ER changes shape in response to cellular cues, cell type, cell cycle state and during development of the organism. Here we discuss what is known about the dynamics of the ER, what questions remain, and how coordinated responses add to the layers of regulation in this dynamic organelle.

## Introduction

The ER is the largest organelle in the cell and is a major site of protein synthesis and transport, protein folding, lipid and steroid synthesis, carbohydrate metabolism and calcium storage [1–7]. The multi-functional nature of this organelle requires a myriad of proteins, unique physical structures and coordination with and response to changes in the intracellular environment. Work from a variety of systems has revealed that the ER is composed of multiple different structural domains, each of which is associated with a specific function or functions. However, it is not yet clear how these functional subdomains are organized and how different functional domains translate into different structures.

## Protein synthesis and folding

One of the major functions of the ER is to serve as a site for protein synthesis for secreted and integral membrane proteins [8], as well as a subpopulation of cytosolic proteins [1]. Protein synthesis requires localization of ribosomes to the cytosolic face of the ER, and the canonical pathway that regulates protein synthesis involves co-translational docking of the mRNA:ribosome complex on the ER membrane. Translation of secretory or integral membrane proteins initiates in the cytosol, then ribosomes containing these mRNAs are recruited to the ER membrane via a signal sequence within the amino terminus of the nascent polypeptide that is recognized and bound by the signal recognition particle (SRP) [9, 10]. The complex of mRNA:ribosome:nascent polypeptide:SRP is targeted to the ER where it docks on the SRP receptor [11, 12]. Translation continues on the ER and the emerging polypeptide can co-translationally enter the ER through the translocon [2], which is a channel that contains several Sec proteins and spans the lipid bilayer [13].

Also during this time, or in some cases once translation is complete [3], a signal peptidase cleaves the short signal peptide allowing the free protein to enter the ER lumen [14]. If the protein is destined to be an integral membrane protein, determined by the presence of a stretch of hydrophobic residues or stop-transfer membrane anchor sequence, translocation will pause [15]. At this point the protein will be shifted laterally and become anchored within the phospholipid bilayer where it remains [15]. Transmembrane proteins can either contain one hydrophobic stretch of amino acids, and are classified as single pass transmembrane proteins, or contain multiple regions that cross the membrane and are classified as multi-pass transmembrane proteins [3].

If the protein is not destined to be integrated into the membrane, but instead enter the secretory pathway or the lumen of membrane-bound organelles, the protein begins the process of transport. Once translation is complete and the signal peptide has been cleaved the ribosomes are released back into the cytosol [16, 17]. For mRNAs translated by stably-bound ER ribosomes, mRNAs are released and ribosomes may remain bound to the ER and participate in multiple rounds of translation [18, 19]. For cytosolic proteins translated on ER-bound ribosomes it is not clear how these mRNAs are recruited to the ER or what populations of ribosomes are utilized to initiate translation, although a recent study indicates that the ER-resident protein p180 may play a role in the translation-independent recruitment of mRNAs to the ER [20].

Following protein synthesis and translocation into the ER lumen, a protein destined for secretion must undergo proper folding and modifications, with the aid of chaperones and folding enzymes. These modifications include N-linked glycosylation, disulfide bond formation and oligomerization [3]. At this point the fate of the secretory proteins is determined. If the protein functions in the ER, for example as a chaperone, then proper folding will commence. If the protein is destined for secretion, it will be released by the chaperones and packaged for travel through the Golgi on to a final destination (such as the plasma membrane or secreted) or move into peroxisomes [21]. Additionally, the cytosolic regions of the transmembrane protein may interact with cytosolic proteins or chaperones to properly fold these domains.

On the other hand, even with several proteins and complexes dedicated to folding proteins properly, a fraction of proteins do not achieve native and functional form and are either misfolded or aggregated [22]. These proteins can either remain in the ER or enter the ER-associated degradation (ERAD) pathway mediated by the proteasome, assuring that aberrant polypeptides do not inadvertently enter the secretory pathway [23]. Recognition of misfolded proteins, followed by clearing of these aggregates through the ERAD pathway, needs to be tightly controlled so as not to affect cellular function [23]. Interestingly, there are several connections to activation of ER stress response pathways and pathological human conditions. Several neurodegenerative protein misfolding diseases, such as Alzheimer’s disease, activate ER stress response pathways. Additionally, activation of the ER stress response pathway is observed in diabetes, inflammatory bowel disease, and various cancers. How ER stress response pathways play a role in these pathologies is an active area of research and various components of the stress response pathways are being investigated as potential therapeutic targets [24]. In general, the protein synthesis functions of the ER are confined to ER sheets and regulation of ER structure by RNA localization and ER stress will be covered later in this review.

## Lipid biogenesis

While the ER is a major site of protein synthesis, it is also a site of bulk membrane lipid biogenesis [4], which occurs in the endomembrane compartment that includes the ER and Golgi apparatus. Proteins and phospholipids, which are the major lipid component of membranes, are transferred and biochemically modified in the region of the ER that is in close juxtaposition to the Golgi apparatus [25]. This region, known as the ER-Golgi intermediate compartment (ERGIC), is rich in tubules and vesicles [4]. Once lipids are mobilized to the ERGIC they are distributed throughout the cell through organelle contacts or secretory vesicles [26]. The *cis*-Golgi, which is the closest structure to the ERGIC, leads to the *trans*-Golgi network where vesicles carrying newly synthesized secretory proteins from the ER form and bud [4]. The *trans*-Golgi network has traditionally been viewed as the main sorting station in the cell where cytosolic cargo adaptors are recruited to bind, indirectly or directly, and transport proteins or lipids [27].

## Calcium (Ca^2+^) metabolism

Finally, while the ER is a major site of synthesis and transport of a variety of biomolecules, it is also a major store of intracellular Ca^2+^ [28, 29]. The typical cytosolic concentration of Ca^2+^ is ~100 nM, while the Ca^2+^ concentration in the lumen of the ER is 100–800 μM, and the extracellular Ca^2+^ concentration is ~2 mM [6, 30]. The ER contains several calcium channels, ryanodine receptors and inositol 1,4,5-trisphosphate (IP_3_) receptors (IP_3_R) that are responsible for releasing Ca^2+^ from the ER into the cytosol when intracellular levels are low [6]. Ca^2+^ release occurs when phospholipase C (PLC) is stimulated through G protein-coupled receptor (GPCR) activation [31] and cleaves phosphatidylinositol 4,5 bisphosphate (PIP_2_) into diacyl-glycerol (DAG) and IP_3_, which can then bind the IP_3_R leading to Ca^2+^ release and transient increase in intracellular Ca^2+^ levels [6]. Ryanodine receptors (RyRs) act through Ca^2+^-induced Ca^2+^ release (CICR), when the receptors bind Ca^2+^ in response to increased cytoplasmic levels of Ca^2+^ [32]. In addition, depolarization of t-tubule membranes can lead to conformational changes in voltage-dependent Ca^2+^ channels, such as dyhydropyridine receptors (DHPRs), which interact and activate RyRs leading to Ca^2+^ release [33]. Furthermore, Ca^2+^ can leak from the ER into the cytoplasm only to be pumped back into the ER via sarcoendoplasmic reticular Ca^2+^ ATPases (SERCAs), or can enter the cell from the extracellular media, adding to the layers of regulation [6]. If ER stores of Ca^2+^ are rapidly depleted through IP_3_ receptor (IP_3_R)-mediated release a mechanism for Ca^2+^ entry into the cell is activated, known as store-operated Ca^2+^ entry (SOCE) [6, 34]. After ER luminal Ca^2+^ depletion, STIM1 proteins cluster in regions of ER abutting the plasma membrane. At these regions, clustered STIM1 traps plasma membrane-diffusing Orai1 subunits [35, 36] and assembles them into active Ca^2+^ release-activated channels (CRAC) allowing for uptake of extracellular Ca^2+^ into the ER lumen to restore Ca^2+^ levels [37–39]. Interestingly, SOCE and activation of CRAC does not depend on, nor sense, changes in levels of Ca^2+^ in the cytoplasm [6], but senses and responds to changes in luminal Ca^2+^ concentration.

Calcium is a widespread signaling molecule that can affect diverse processes including localization, function and association of proteins, either with other proteins, organelles or nucleic acids. Release of Ca^2+^ can result in a wave of Ca^2+^ that moves through the entire cell [40], a gradient of Ca^2+^ from the source of release, or a spatially-restricted wave from clustered channels known as a Ca^2+^ spark [41]. One of the most well-studied Ca^2+^ release events occurs at fertilization following sperm entry [40, 42], but also occurs during muscle contraction and secretion [6] as well as neuronal processes including neurotransmitter release [43]. We will highlight recent evidence that Ca^2+^ may also play a role in reshaping the ER in response to cellular signals.

## Regulation of ER shape and function

**Fig. 1:**
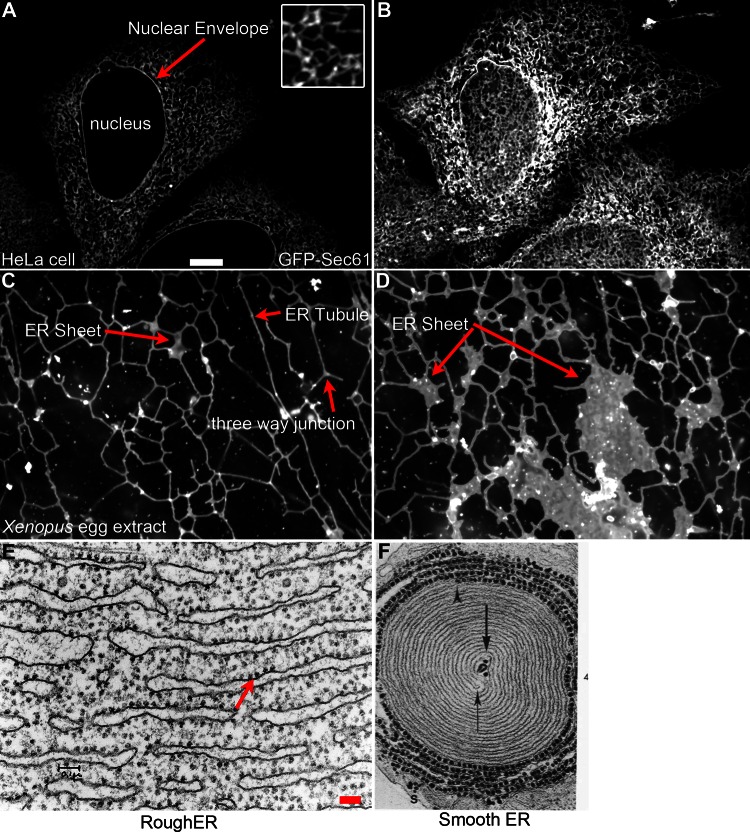
Various ER structural morphologies. **a** Location of the ER visualized in a HeLa cell transfected with GFP-Sec61β. *Inset* shows the polygonal network of the peripheral ER magnified ×3 relative to the magnification in **a**. This view highlights the relationship of the ER to the nuclear envelope (*red arrow*). **b** ER morphology from the same HeLa cell depicting an image plane closer to the coverslip. This highlights the complexity of the peripheral ER. **c** ER network formed in *Xenopus* egg extracts. Three-way junctions, ER tubules and small ER sheets are highlighted (*red arrows*). **d** ER network formed in *Xenopus* egg extracts highlighting large ER sheets containing ribosomes (*red arrow*). *Scale bar* for **a**–**d** is 10 μM and is shown in **a**. **e** Electron micrograph (EM) of rough ER from guinea pig pancreas. Reprinted with permission from James Jamieson. *Scale bar* is 0.1 μM. **f** EM of smooth ER from ocular rabbit muscle. Reprinted with permission from Fig. 4 [164]. Magnification is ×50,000

The ER is a complex organelle, involved in protein and lipid synthesis, calcium regulation and interactions with other organelles. The complexity of the ER is reflected in an equally complex physical architecture. The ER is composed of a continuous membrane system that includes the nuclear envelope (NE) and the peripheral ER, defined by flat sheets and branched tubules (Fig. 1). The shape and distribution of these ER domains is regulated by a variety of integral membrane proteins and interactions with other organelles and the cytoskeleton. These interactions are dynamic in nature and reflect changes within the cell, either through cell cycle or developmental state, cell differentiation, intracellular signals or protein interactions. While it is generally known how the basic shapes of ER sheets and tubules are determined, it is relatively unclear how changes in shape or the ratio of sheets to tubules occur in response to specific cellular signals.

Here, we will discuss what is known about how the structures of ER are formed, how the dynamics of the ER are regulated, and how these dynamics change in response to cell cycle state and cellular cues. In addition, we provide examples of how the proteins that are involved in contributing to ER shape are influenced by these cellular cues, such as calcium release, and how this is reflected in the dynamics of the ER and ultimately the function of specialized cells that display varying ratios of sheets to tubules.

## ER structure

There have been several excellent, recent reviews that cover the topic of general ER structure in detail [7, 44–48], so we will limit our review of the basic ER structure to only those factors that may play a role in changing the shape of ER in response to signaling. The ER consists of the nuclear envelope and the peripheral ER, which includes smooth tubules and rough sheets. While the ER is defined as an interconnected network with a continuous membrane, the different structures that make up the ER perform very diverse and specialized functions within the cell.

**Fig. 2:**
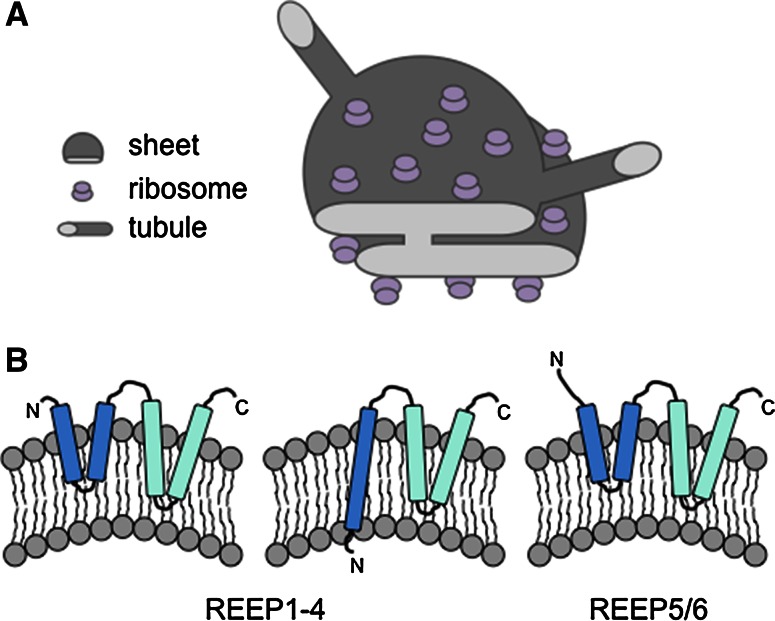
Structure of ER sheets and tubules. **a** ER sheets and tubules have a diameter of 30–50 nm in eukaryotes. Eukaryotic ribosomes are 25–30 nm and localize to the flat regions of ER sheets, giving the sheets a rough appearance (rough ER). Ribosomes are present in much lower numbers on tubules, giving the tubules a more smooth appearance (smooth ER). **b** Models of potential hairpin topologies of REEP family proteins that act as wedges to promote bending of the membrane, adapted from [63]

The nuclear envelope is made up of two lipid bilayers, the inner nuclear membrane (INM) and outer nuclear membrane (ONM), and shares a common lumen with the peripheral ER. Hundreds of nuclear pores spanning the ONM and INM of the nuclear envelope allow transport of molecules, including RNAs and proteins, at various rates of diffusion or regulated transport depending on the size of the molecule. The nuclear envelope is connected to sheets, or cisternae, that make up part of the peripheral ER. Sheets are flat in nature consisting of two lipid bilayers with an intervening lumen, with curved regions located only at the membrane edges. Peripheral ER Sheets may vary in size, but the luminal spacing is very consistent, usually about 50 nm in mammals and 30 nm in yeast [49] (Fig. 2). Sheets are usually observed in a stacked conformation and are connected via regions of twisted membranes with helical edges [50]. These rough sheets, as defined by the high density of ribosomes on the cytosolic surface [51, 52], are the main site of synthesis, folding and post-translational modifications for secreted or membrane-bound proteins. In turn, far fewer ribosomes are present on the membrane surface of ER tubules [52], which is highly curved and smooth and may not accommodate the binding of large polysomes (Fig. 2). The tubular network is dynamic, continually rearranging and growing, and is defined by three-way junctions that connect individual tubules (Fig. 1). While tubules and sheets possess very different structural features, and hence play a role in different cellular processes, the luminal spacing of both tubules and sheets is similar [49, 52].

Interestingly, ER tubules and sheets are found in all eukaryotic cells [53], though the ratio of sheets to tubules varies in different cell types and reflects the different functions of these cells. For example, the ER architecture of specialized cells that synthesize vast amounts of secreted proteins, such as pancreatic secretory cells and B cells, is largely made up of sheets (Fig. 1). In turn, cells that are involved in processes including lipid synthesis, calcium signaling and sites of contact for other organelles possess an ER composed of primarily tubules (Fig. 1). Adrenal, liver and muscle cells are all examples of specialized cells with a predominantly tubular network and reflects the function of these cells [54].

An additional configuration of the peripheral ER includes cortical ER, which abuts the plasma membrane and displays an intermediate phenotype between sheets and tubules with membranes that are both highly curved as well as regions that are flat in nature. Calcium signaling occurs at the contact sites between the plasma membrane and the abutting cortical ER and is necessary for muscle contraction [55, 56]. Therefore, the morphology and intracellular location of the ER subdomains contribute to the function of these structures and hence the role of the specialized cell in which they are located.

Improved microscopy techniques have allowed for the characterization of different ER structures, and the ratios of these structures to one another, in specialized cell types. When comparing the roles of these cells in the organism, it is clear that the type and amount of peripheral ER present reflects the function of that particular cell type. It is still unclear how these ratios are generated and what cellular signaling pathways play a role in designating which ER type will be most prominent in a particular cell type.

## ER shaping proteins

### ER tubules

Peripheral ER structures are just as distinct and diverse as the set of proteins that contribute to their shape. Several proteins have been identified that promote specific ER structures, but perhaps the most well-studied group of proteins include the reticulon family of proteins that localize to tubules and the highly curved edges of ER sheets [51, 57]. These integral membrane proteins contribute to the bending of the membrane by forming a transmembrane hairpin topology that acts as a wedge, displacing lipids in the outer leaflet of the bilayer leading to curvature of the membranes [57]. These proteins tend to form oligomers and are much less mobile than other ER-resident proteins [58]. Overexpression of some reticulon isoforms leads to formation of long ER tubules at the expense of sheets [58]. In turn, depletion of reticulons, and hence the ability to bend membranes, leads to a reduction in the number of ER tubules, leading to an expansion of peripheral sheets [57, 59, 60]. Therefore, the level of reticulons within a cell determines the abundance and fine structure of ER tubules.

Reticulons do not act alone in shaping ER tubules. Members of the DP1/Yop1/REEP5/6 and REEP1-4 family, which are abundant ER-resident proteins that specifically localize to tubules and edges of sheets, also act as tubule-promoting factors. DP1/Yop1, or REEP5/6 [61], proteins share a similar transmembrane hairpin architecture with the reticulons (Fig. 2), leading to the stabilization of the curved membranes of tubules [57, 58, 62]. Interestingly, REEP1-4 proteins have a topology distinct from REEP5/6 suggesting that these proteins may have slightly different functions in shaping the ER than the closely related REEP5/6 proteins [63] (Fig. 2). Additionally, purified reticulons and DP1/Yop1 family proteins were able to induce tubule formation from purified vesicles [62], demonstrating that these proteins play an essential role in ER tubule growth.

Reticulons and DP1/Yop1 promote tubule formation, but additional factors are required to promote the formation of the tubular network and characteristic three-way junctions through homotypic fusion. Atlastins, members of the dynamin-like GTPase family, mediate these homotypic fusion events. Depletion by RNAi or expression of dominant-negative atlastin in cells results in a lack of fusion events leading to an abundance of long, unbranched tubules [61]. When a dominant-negative cytoplasmic fragment from *Xenopus*, which contains the GTPase domain but lacks the transmembrane domain and cytoplasmic tail [64], are introduced into *Xenopus* interphase extracts ER network formation was blocked [65]. Comparable point mutations that prevent dimerization of the cytoplasmic fragment of human atlastin [66] were made in the *Xenopus* cytoplasmic atlastin protein, added into interphase extract and had no effect on ER network formation [65]. Furthermore, antibodies directed against atlastin inhibit ER network formation when introduced into *Xenopus* egg extracts [61]. In *Drosophila*, atlastin depletion leads to ER fragmentation and purified atlastin is sufficient to catalyze GTP-dependent fusion of proteoliposomes [64, 66, 67]. Therefore, studies from multiple organisms, extracts and purified components indicate that atlastin is likely required for catalyzing homotypic vesicle fusion between ER membranes, which is important for proper network formation.

Recently, a few new key players have been identified that are involved in ER dynamics. Work using purified ER vesicles derived from *Xenopus* eggs has demonstrated that GTP is required for homotypic ER vesicle fusion in the absence of cytosolic factors [57, 68]. Previous studies indicated that GTPases are required for ER fusion events [69, 70], and a recent study utilized a proteomics approach to identify Rab10 as a factor required for ER assembly [71]. Knock-down of Rab10, or overexpression of a GDP-locked dominant-negative point mutant, in cultured human cells caused an increase in ER sheets and a decrease in tubules [71]. ER–ER fusion events occurred at regions where Rab10 was enriched. Rab10 was found to co-localize with several lipid-synthesizing enzymes, including phosphoinositol synthase (IS) and choline/ethanolamine phosphotransferase (CEPT1) [71], leading to the possibility that this may represent a previously unidentified ER subdomain or compartment. It is currently not clear what role Rab10 plays in the ER vesicle fusion reaction or how homotypic ER vesicle fusions are coupled to lipid synthesis.

Recent work has also identified a role for Rab18, which is targeted to the ER by Rab3 GTPase activating protein (GAP) complex, in ER dynamics. Depletion of Rab18 leads to a phenotype similar to that observed following Rab10 inhibition [72]. Additionally, when Rab10 is depleted, Rab18 redistributes to peripheral sheets [72]. Therefore, it appears that depletion of either Rab10 or Rab18 prevents the stabilization of ER tubule fusion, reducing the density of tubules resulting in an increase in ER sheets. Depletion of the *Caenorhabditis elegans* RAB-5, which has been previously implicated in early endosome function [73], phenocopies the peripheral ER defects seen in the RET-1 and YOP-1 (homologs of Rtn4a and DP1) depletions [70]. In addition to the role RAB-5 plays in peripheral ER formation, kinetics of nuclear envelope disassembly is affected in these mutants [70].

In addition to GTPases that may play a direct role in homotypic membrane fusion of vesicles, recent work has demonstrated a role for lipid synthesizing enzymes in controlling the shape and organization of the ER. Inhibition of C-terminal domain (CTD) nuclear envelope phosphatase-1 (CNEP-1), which is enriched on the nuclear envelope and promotes the synthesis of membrane phospholipids, led to the appearance of ectopic sheets that encased the nuclear envelope, interfering with nuclear envelope breakdown [74]. These results reflect the interconnected network of proteins and functions that play a role in shaping the structures of the ER.

The ER is a very dynamic network that is constantly undergoing rearrangements and remodeling [75]. ER tubules are continually fusing and branching resulting in the creation of new three-way junctions. In a competing process, junction sliding and tubule ring closure leads to loss of three-way junctions and the characteristic polygonal structure [76]. Very little is known about the complexes controlling this process, but it was recently discovered that Lunapark (Lnp1) localizes to and stabilizes three-way junctions [77, 78]. Lnp1 binds to reticulons and Yop1, and localization of Lnp1 to junctions is regulated by Sey1p, the yeast homolog of atlastin [78]. Loss of Lnp1 leads to a collapsed and densely reticulated ER network in yeast and human cultured cells [77, 78], though only half of the junctions are bound to Lnp1 [77], which reflects the fluidity of the ER network. If Lnp1 is overexpressed, the protein localizes to the peripheral ER and induces the formation of a large polygonal tubular network [79]. Additionally, formation of this network was inhibited by Lnp1 mutations that blocked *N*-myristoylation [79], an attachment of myristic acid (a 14-carbon saturated fatty acid), indicating that this modification plays a critical role in Lnp1-induced effects on ER morphology. *N*-myristoylation is not required for membrane translocation, topology formation, or protein localization to the ER but may play a role in protein–protein or protein-lipid interactions that are required for morphological changes in the ER, though the exact molecular mechanism of action remains to be elucidated [79].

The actual mechanism for Lnp1-mediated stabilization of three-way junctions is unknown, though recent studies and insights from the structure and domains within the protein shed light on how Lnp1 stabilizes junctions [77, 78]. First, Lnp1 contains two transmembrane domains as well as a zinc finger domain, which is located on the cytoplasmic face of the ER membrane [77]. When cysteines were mutated within the zinc finger domain, the polygons became smaller and regions lacking cortical ER were more apparent as the number of cysteines mutated increased [78]. Therefore, mutations in the zinc finger domain may affect protein–protein interactions, complex formation or interfere with the distribution of resident lipids on the cytoplasmic face of the membrane causing deleterious effects on junction stabilization. In addition, the transmembrane domains may be acting as an inverted wedge, adding to the local negative curvature characteristic of three-way junctions [77], and acting opposite to the positive curvature promoted by reticulons. Another possibility is that multiple Lnp1 proteins may also act cooperatively together to stabilize the junction, or Lnp1 may be acting transiently to stabilize or modify lipids or other proteins at junctions [77].

In addition to proteins that regulate membrane structure and dynamics, there is accumulating evidence that changing the nucleic acid content of the ER can also impact ER shape. Early experiments showed that brief treatment of tissue culture cells with the translation inhibitor puromycin, which dissociates mRNA:ribosome complexes, leads to loss of ribosomes from the ER and a loss of ER sheets [51, 80]. This suggests that the presence of mRNA:ribosome complexes may stabilize ER sheets. In support of this hypothesis, our recent work identified an ER-localized ribonuclease, XendoU [81], that changes the RNA content of the ER in response to changes in free Ca^2+^ concentration [82, 83]. These changes occur at physiologically relevant levels of ~1.5 μM, which mimics release of Ca^2+^ from intra- and extracellular stores at fertilization [42, 84]. A subpopulation of XendoU localizes to the ER and co-immunoprecipitates with a number of ER-resident proteins [82]. Depletion of XendoU leads to the formation of long, unbranched tubules in *Xenopus leavis* egg extract, and rescue of this phenotype requires intact catalytic activity of the protein, indicating that the nuclease function is critical to proper ER network formation [82]. Furthermore, antibody addition to purified vesicles leads to a block in network formation, demonstrating that XendoU acts on the surface of ER membranes to regulate ER structure [82]. Interestingly, addition of 5′5′-dibromo BAPTA, a strong calcium chelator, blocked vesicle fusion in this system [68]. Depletion of XendoU also leads to a delay in replication and nuclear envelope closure [82], and BAPTA blocks nuclear envelope formation in *Xenopus* egg extract reconstitution experiments [85]. Together these results suggested that XendoU acts on membranes to degrade RNAs.

Upon vesicle fusion it was found that RNAs were degraded and released from the surface of membranes, suggesting that XendoU acts to degrade these RNAs, as well as release proteins, to clear patches of membrane to allow for vesicle formation leading to network formation [82]. Interestingly, when purified vesicles were treated with increasing concentrations of RNaseA and subjected to the same assay, an increasingly aberrant network formed with large vesicles that were unable to fuse [82]. Results from in vitro studies indicate that XendoU is activated on membranes in coordination with calcium release to locally degrade RNAs and clear patches of membranes leading to fusion in a controlled manner to fine tune network formation.

Lastly, similar to other proteins that play a role in tubule formation, knock-down of the human homolog EndoU in cultured human cells leads to an expansion of sheets [82]. Additionally, rescue of the expanded sheet phenotype depended on intact catalytic function as observed with recombinant protein in the extract system. Therefore, XendoU is an example of a protein that is activated in response to cellular cues to regulate proper ER formation, and further studies may reveal additional proteins that are regulated in this manner to fine tune organelle structure.

### ER sheets

We have considered how tubules are formed and maintained, which leads the discussion to sheets, the other peripheral ER structure. First, we must consider how sheets are formed. Several mechanisms have been proposed, including the idea that integral membrane proteins can span the intraluminal space and form bridges, connecting the lipid bilayers [51, 86, 87]. These proteins may either stabilize the structure or define the distance between the two lipid layers based on the size of the proteins. Additionally, these proteins or protein complexes may form a scaffold that aids in the stabilization of the sheets or bring the two lipid membranes in closer proximity [86]. Several proteins including Climp63, p180 and kinectin have been implicated in the generation, maintenance and stabilization of ER sheets [51].

In addition to highly enriched membrane proteins and core components of the translocon, Climp63, a coiled–coiled protein with a single transmembrane domain, was identified along with kinectin and p180 in a mass spectrometry screen for abundant integral ER membrane proteins [51]. Through various techniques and in various cell types Climp63 was shown to be a highly abundant protein [88–90] that localizes to perinuclear ER and is absent from the nuclear envelope [91, 92]. Very stable oligomers of Climp63 can form, restricting mobility of the protein along the membrane, promoting localization to the rough ER [92]. Overexpression of Climp63 leads to a massive proliferation of ER sheets while reduction in expression surprisingly does not lead to loss of sheets but instead a decrease in the distance between sheets [51]. Moreover, these sheets are spread diffusely throughout the cytoplasm, reminiscent of the phenotype of cells treated with the translation inhibitor puromycin [51]. This is interesting as the core components of the translocon, the protein channel that interacts with ribosomes and is responsible for translocating nascent peptides into the ER or anchoring transmembrane segments of newly synthesized proteins, were found to be enriched on sheets [93]. Therefore, these results suggest that the role of Climp63 in formation of sheets is likely to involve additional factors and acts as a part of an elaborate regulatory network that balances the production of sheets and tubules.

### ER microtubule interactions

It is clear that proteins involved in the promotion, maintenance or stabilization of peripheral ER structures function through interactions with additional proteins or structures, and these interactions are key to proper formation of the ER network. Interestingly, several of the proteins discussed above have been shown to interact with microtubules, including Climp63 [91], p180 [94], kinectin [95] and STIM1 (discussed below). One important interaction discussed below is with microtubules. The ER network exhibits several dynamic interactions with microtubules that are important for determining the distribution of the ER within the cell. The two main types of interactions between the ER and microtubules are Tip Attachment Complexes (TACs) and sliding along preformed microtubules by the action of kinesin and dynein motors [96–100]. In cultured cells treated with nocodazole to depolymerize microtubules, the ER retracts from the periphery [101], though the retraction does not occur immediately. Further investigation revealed that sliding events occurred mainly on a small subset of microtubules, modified by acetylation, that are more resistant to nocodazole treatment [76]. Furthermore, ER tubules can form in the absence of microtubules [57, 65, 68], raising many questions and leading several groups to study the interaction between ER and microtubules more in-depth.

In the past 10 years we have learned a great deal about what proteins are responsible for the intrinsic shape of the ER and how these proteins are connected to specific ER subdomains. However, we know very little about how cellular signals communicate with ER shaping proteins to change the shape of the ER in response to cellular signals.

### Changes in ER structure during mitosis

During mitosis many cellular structures are dramatically remodeled to facilitate chromosome segregation. One of the most dramatic examples is changes to the microtubule cytoskeleton that occur as a result of increased microtubule dynamics caused by the action of cyclin-dependent kinases. The increase in microtubule dynamics during mitosis is important for the bipolar attachment of chromosomes to the mitotic spindle and accurate segregation to daughter cells during anaphase [102]. In addition to changes to the microtubule cytoskeleton, essentially all organelles change shape and function during mitosis to facilitate accurate organelle inheritance and orderly chromosome segregation. The ER undergoes dramatic shape changes during mitosis and recent studies are beginning to uncover the mechanisms linked to these structural changes.

In organisms with an open mitosis the nuclear envelope breaks down at the onset of mitosis to allow free exchange between the nucleus and cytoplasm. Nuclear envelope breakdown (NEBD) is a carefully orchestrated process that begins during mitotic prophase [103]. During prophase components of the nuclear pore dissociate from the pore, the nuclear lamina depolymerizes, and the membrane-bound proteins of the nuclear envelope retract into the general ER. These events free the chromosomes of nuclear lamina and membranes to facilitate chromosome condensation and segregation. In general, the events of nuclear envelope breakdown are thought to be driven by the phosphorylation of components of the NE during mitosis by various mitotic kinases, especially cyclinB:cdk1, although many molecular details are still unclear.

Concomitant with changes that occur to the nuclear envelope during NEBD the ER also begins to undergo dramatic shape changes. Changes in ER shape during mitosis have been studied in many different organisms by both light and electron microscope and these studies have resulted in a conflicting series of reports about the shape of the ER during mitosis. However, during the last few years a consensus has begun to emerge that the mitotic ER is primarily composed of sheets. Early studies using live cell microscopy in both *Drosophila* and *C. elegans* embryos demonstrated that the ER changed from a mixture of sheets and tubules to almost exclusively sheets during mitosis [104, 105]. Additionally, work using thin section transmission EM in HeLa cells also concluded that the majority of the ER was present in sheets throughout mitosis [106]. However, two studies in a variety of mammalian tissue culture cells [80, 107] have used both live cell microscopy and electron microscopy to suggest that the ER is primarily tubular during mitosis, and two additional studies [60, 108] also suggested that the ER remained tubular during mitosis and further suggested that end-on binding of ER tubules to chromatin during mitosis initiates nuclear envelope reassembly at the end of mitosis. One potential difficulty in interpreting the shape of the mitotic ER is that most cells round up during mitosis which can make acquisition of light and electron microscopy images difficult and require laborious reconstruction of the images into a three dimensional model. In addition, the mitotic ER is highly dynamic, which can complicate acquisition of live cell images during mitosis. To address these questions a series of recent studies have used both high-resolution, high-speed live cell microscopy and high-resolution EM to demonstrate that the ER is almost exclusively composed of sheets during mitosis [109, 110]. In addition, these studies demonstrate that the nuclear envelope reforms through the docking of ER sheets onto regions of chromatin that are isolated from spindle microtubules [109]. Finally, to circumvent many or the problems associated with imaging large, three dimensional cells during mitosis a recent study has examined the structure of the ER in vitro using ER reconstituted from *Xenopus* egg extracts [65]. This study convincingly demonstrated that ER formed in mitotic extracts is primarily composed of sheets while interphase ER is primarily composed of tubules. In addition, the authors demonstrated that active cyclinB:cdk1 was sufficient to convert a tubular ER into a primarily sheet based ER. Taken together all of these studies present conflicting views of the shape of the ER during mitosis, but a consensus is emerging from a wide variety of organisms that the mitotic ER is primarily composed of sheets and that the shape changes in the ER are related to changes in cyclin:cdk activity.

In addition to changes in the gross morphology of the ER during mitosis there are also dramatic changes in the distribution of proteins throughout the ER. During interphase the ER is organized into distinct domains with certain proteins defining different domains. For example, the tubule-shaping reticulon protein Rtn4 is exclusively present in the peripheral ER and excluded from the nuclear envelope [57, 60, 110]. In contrast, some proteins, such as the Lamin B receptor and components of the nuclear pore, are exclusively present in the nuclear envelope and are excluded from the peripheral ER [60, 110], while some proteins, like Sec61β, are present in all ER subdomains. However, during mitosis the NE retracts into the ER and there is nearly complete mixing of the specialized ER-shaping proteins [60, 110]. At the end of mitosis proteins that define the NE and peripheral ER are rapidly resorted such that they reestablish their characteristic interphase organization [60, 110]. In addition, it has been shown that overexpression of Rtn4 or knockdown of three reticulons (Rtn1, Rtn3, Rtn4) can either slow or speed the rate of NE reassembly at the end of mitosis, although the mechanism through which these proteins affect NE formation is currently unknown. These studies highlight the massive reorganization that takes place in the ER during mitosis and suggests that different expression levels of specific ER shaping proteins can control ER reorganization during mitosis. However, we know very little about how various ER shaping proteins are resorted to specific domains at the end of mitosis.

Two very recent studies [111, 112] have begun to provide insight into the specialized processes that regulate nuclear envelope reformation at the end of mitosis. Both of these studies identified a transient localization of the ESCRT-III complex to the surface of chromatin during late anaphase when the nuclear envelope is beginning to reform. ESCRT-III is best known for its role in the formation of multivesicular bodies during endocytosis, but also has well-documented roles in cytokinesis and viral budding from the plasma membrane [113]. Both studies demonstrated that the membrane binding and deformation properties of ESCRT-III are required for nuclear envelope formation. Additionally, interactions with the microtubule severing enzyme spastin and the ubiquitin recognition factor UFD1 are important for nuclear envelope reformation. These results demonstrate that an endosomal complex is important for regulating NE reformation and suggest that ESCRT-III could potentially play a role in additional aspects of ER dynamics.

The redistribution of ER shaping proteins during mitosis suggests that the fundamental activities of some of these proteins are modified during mitosis. For example, the mitotic ER is composed of primarily sheets, yet Rtn4, which promotes tubule formation [57], is distributed throughout the ER [60, 110]. This result suggests that the tubule-promoting activity of Rtn4 may be modified during mitosis to facilitate the tubule-to-sheet transition observed during mitosis. Inspection of large-scale phospho-proteomics studies reveals that a large number of ER-shaping proteins have identified mitosis-specific phosphorylation sites [114–121]. Although none of the phosphorylation sites identified in these large-scale screens has been studied in detail their presence and specificity to mitosis suggests that these are likely to be involved in reshaping the ER during mitosis.

In support of the hypothesis that mitosis-specific phosphorylation of ER-shaping proteins regulates ER remodeling during mitosis two studies have examined this phenomenon in detail. A study of the ER sheet promoting protein Climp63 [51] has demonstrated mitosis-specific phosphorylation on three N-terminal residues [121]. Phosphorylation of Climp63 blocks the interaction of Climp63 with microtubules. Additionally, phosphomimetic mutants blocked the interaction of the ER with microtubules during interphase and resulted in an ER composed primarily of sheets, while nonphosphorylatable mutants tethered the ER to microtubules and resulted in an extremely distorted ER. These results suggest that mitotic phosphorylation of Climp63 likely blocks the interaction of the ER with microtubules and could be an important step in the tubule-to-sheet transition that occurs during mitosis. A second study examined the interaction of the ER with growing microtubule plus ends during mitosis. During interphase the ER-associated protein STIM1 interacts with the microtubule plus end-binding protein EB1 to couple ER reshaping to microtubule polymerization [122]. However, during mitosis the ER is excluded from the mitotic spindle and does not exhibit plus tip growth events. A recent study [123] has demonstrated that STIM1 is specifically phosphorylated during mitosis to control the interaction of the ER with microtubules. Specifically, phosphorylation of STIM1 blocks the interaction with the plus-end tracking protein EB1. Nonphosphorylatable mutants of STIM1, created by mutation of 10 S/T residues that block all mitotic phosphorylation, result in a recruitment of the ER throughout the spindle by restoration of the interaction of STIM1 with EB1, demonstrating that phosphorylation is a major mechanism that regulates the association of the ER with microtubules during mitosis. Interestingly, phosphorylation of STIM1 also blocks activation of SOCE, although this occurs independently of the STIM1:EB1 interaction [118]. Clearly much more work remains before we have a clear understanding of how cell cycle signaling cascades contribute to reshaping of the mitotic ER.

While the above studies demonstrated that phosphorylation of key proteins that link the ER to the microtubule cytoskeleton is important for excluding the ER from the spindle during mitosis a recent study demonstrated the importance of an interaction of the ER with microtubules for clearing the ER from mitotic chromatin. During mitosis the nuclear envelope is absorbed into the ER and is cleared from the surface of the chromatin, however little is known about the mechanisms that regulate ER removal from the chromatin. A recent study used a biochemical approach to identify proteins that bind to both membranes and microtubules to identify new ER proteins REEP3/4 [124]. The authors demonstrate that RNAi against REEP3/4 results in a failure to remove membranes from chromosomes during mitosis, resulting in chromosome segregation defects and internuclear membrane inclusions. Interestingly, the authors further demonstrate that removal of membranes from mitotic chromatin requires the interaction of REEP3/4 with microtubules. However, it is not known if REEP3/4 is subject to phosphoregulation during mitosis or if the microtubule-binding activity or REEP3/4 is required for shaping the ER during interphase. Taken together these three studies demonstrate that interaction of the ER with microtubules is a major mechanism that contributes to shape rearrangement during mitosis and that ER:microtubule interactions are regulated by mitotic phosphorylation. In addition, these studies demonstrate that the ER interacts with microtubules using many different adaptor proteins and that these different adaptor proteins serve different functions during mitosis.

### Changes in ER during oocyte maturation and fertilization

One of the greatest changes during development occurs at fertilization. As in mitosis, the transition from oocyte to embryo requires many coordinated cellular changes including release from meiotic arrest, resumption of mitosis, fusion of pronuclei, activation of signaling cascades and changes in protein expression [125–128]. In order for development to proceed normally, the egg must undergo the proper calcium response in order to initiate the developmental program and embryogenesis [129].

While the exact mechanism and conformational changes vary slightly among all organisms studied, the ER architecture in oocytes of all animals changes including *Xenopus* [130, 131], sea urchin [132], starfish [133] and mouse [134]. Initial studies in starfish oocytes revealed that the ER is comprised of interconnected sheets of membranes, though following germinal vesicle breakdown (GVBD), the ER sheets wrap around yolk platelets resembling a shell [133]. In immature mouse oocytes, large clusters were found deep within the cytoplasm [134]. Following GVBD, the spindle and surrounding ER migrate to the cortex leading to another round of ER reorganization into vegetally localized clusters in the metaphase II egg in addition to a finer reticular network throughout the egg [134, 135]. Interestingly, these steps are dependent on the microtubule network as nocodazole and inhibition of cytoplasmic dynein both prevent the ER reorganization [135]. Formation of the ER clusters is prevented by the depolymerization of microfilaments, but not microtubules [135]. Given the timing of each of these reorganizations, it seems likely that they are related to increases in cyclinB:cdk1 activity that occurs upon oocyte maturation [136]. These observations show an additional time in development where the ER and microtubule network interact to regulate ER structure.

In *Xenopus* immature oocytes, the network in both the animal (pigmented) half and vegetal (unpigemented) half appears to be uniform and consists of tubules and individual, unstacked sheets [130]. Additionally, the vegetal half contains annulate lamellae, stacks of sheets with membranes containing densely packed nuclear pores [130]. In mature eggs, the ER in the animal half is unchanged, however the annulate lamellae in the vegetal half disappeared. Interestingly, it has been proposed that the annulate lamellae share many properties with the nuclear envelope [137]. In place of the annulate lamellae dense, irregularly shaped ER clusters were present. The appearance of these clusters coincided with germinal vesicle breakdown. These clusters disappeared and reappeared throughout maturation and upon fertilization dispersed and permanently disappeared. The reorganization of the ER is coupled to the cell cycle as the clusters present in mature eggs contain IP_3_ receptors [130] and release calcium from IP_3_ channels at fertilization [138, 139].

Along with these changes comes a transient intracellular calcium wave, initiated during sperm entry, released from the ER and extracellular stores [40, 42, 140–142]. There is one major difference in eggs of mice versus eggs of frogs. Frogs, as well as sea urchin [143] and starfish [133, 144] have a single calcium transient at fertilization [145]. Other animals, including mice and humans, have multiple smaller calcium transients following fertilization, and these differences may be reflected in the ER organization in mature eggs [145]. Mice [134] and frogs display ER clusters that are similar in size and location (the side opposite the meiotic spindle) and possess IP_3_ receptors [130, 146]. However, fertilization in mice occurs on the side with the ER clusters whereas fertilization in frogs occurs in the animal pole where the meiotic spindle is located. Therefore, the clusters may be involved in secondary calcium wave propagation. The organization of the ER network, and the reorganization throughout oogenesis, serves as a functional consequence of calcium signaling and propagation in these organisms [129]. We currently do not know much about the molecular mechanisms that lead to changes in ER shape during meiotic maturation and fertilization, and this should be a major are of research interest.

### ER changes in response to ER stress

As seen so far, the ER is an organelle of many different functions that must be tightly regulated to carry out the proper functions. One of the most prominent functions of the ER is protein synthesis. Even with several chaperones and folding enzymes in place, an accumulation of unfolded or misfolded proteins in the lumen of the ER can occur. When the cell undergoes this type of stress there are several things that must occur to retain balance and proper function, including translational inhibition, degradation of unfolded or misfolded proteins, and an increase in the production of chaperones and folding enzymes to restore normal function of the ER and the cell. If the balance is not restored it can lead to cell death or apoptosis [147], therefore achieving normal function is critical to the survival of the cell.

As discussed above, once a peptide destined for secretion has entered the lumen of the cell, there are several modifications that occur, including N-linked glycosylation, disulfide bond formation and oligomerization [3]. N-linked glycosylation can occur co-translationally as the protein is translocated into the ER lumen. The oligosaccharyltransferase (OST) can modify the Asparagine within the Asn-X-Ser/Thr sequence once it has traversed approximately 13 amino acids into the ER lumen [148], which improves the kinetics and thermodynamics of folding for proteins [149, 150]. Misfolding can occur due to the unique environment of the lumen and the high protein concentration of both newly synthesized proteins, proteins ready for secretion and proteins that act as molecular chaperones and folding enzymes. Logistically, due to the high protein concentration and packing in the lumen, the folding enzymes must first identify and find the proper target protein for folding to take place. If proteins are not modified correctly, the lack of glucose residues is recognized by the ER and proteins including UDP-glucose:glycoprotein glucosyltransferase (UGGT) in an attempt to re-glycosylate the protein [151–153]. If the normal folding process is not restored, hydrophobic residues are exposed and bound by Grp78, accumulation of these proteins occurs and the unfolded protein response (UPR) is activated [154, 155]. The first action of the UPR is to increase ER abundance to accommodate the needs of the cell to properly fold the proteins, leading to an expansion of the ER through the generation of sheets [156] and an increase in the ER folding machinery.

The UPR consists of three parallel branches that are activated upon stress and include inositol requiring enzyme 1 (IRE1) by nonconventional splicing, double-stranded RNA-activated protein kinase (PKR)-like ER kinase (PERK) through translational control by phosphorylating eIF2α, and activating transcription factor 6 (ATF6) through regulated proteolysis [155]. Briefly, activation of these pathways lead to production of b-ZIP transcription factors that activate UPR genes [155]. First, ER-resident IRE1, a transmembrane endoribonuclease, mediates the post-transcriptional, non-canonical splicing of XBP1 mRNA that is localized to the ER [157–159] and encodes a transcription factor involved in upregulating additional stress response genes. Additionally, the nuclease activity of IRE1 is involved in degradation of a subset of ER-associated RNAs in a process known as IRE1-dependent decay (RIDD) [160, 161]. The cell has evolved this mechanism to reduce the translational load on the ER by removing mRNAs that otherwise would be translated, and may be one way for the cell to upregulate stress-response genes that are needed in the UPR. Although it is clear that ER-stress leads to large scale changes in the protein and RNA content of the ER, it is not yet clear if this leads to immediate structural reorganization in order to accommodate the new needs of the organelle. In addition, it is not yet clear if activation of stress-responsive signaling pathways leads to the modification of intrinsic structural components of the ER. Interestingly, it has been observed that splicing of XBP1 is activated during meiosis in both *Xenopus* and budding yeast [162, 163], suggesting that changes in ER structure during meiosis could be linked to the ER stress response. These would both be interesting avenues of future research exploring structural changes in the ER in response to cellular signaling cues.

## Closing remarks

The ER is a complex organelle that plays a pivotal role in protein and lipid synthesis, calcium storage and stress response. Changes in structure in response to cell cycle or developmental state render this organelle highly dynamic. Several proteins play a role in the proper formation of the different structures of the peripheral ER including the nuclear envelope, sheets and tubules. Regulation exists at multiple steps in the formation and maintenance of these structures, and the ratios of these structures are very different in cells of different functions. In general, cells involved in synthesizing large amounts of protein have higher ratios of sheets, whereas cells involved in lipid synthesis or signaling with other organelles would have higher ratios of tubules. The generation of these structures relies on a myriad of proteins, involved in either structural aspects of ER morphology by directly affecting the phospholipid bilayer and curvature of membranes or mediating interactions with other organelles or the cytoskeleton. In addition, proteins with other functions, including nucleases and GTPases, also play a role in network formation. Recent work has begun to connect our knowledge of the proteins that provide the fundamental shape of the ER to signaling pathways, but much work remains to be done to understand how developmental, cell cycle, and stress pathways change the fundamental shape of the ER in different circumstances. Recent work on several different human diseases has highlighted a role for several different ER-shaping proteins in diverse diseases such as Alzheimer’s and Hereditary Spastic Paraplegia (HSP) [reviewed in 7]. The strong link of ER-shaping proteins to hereditary human diseases highlights the need for further research into the basic biology of the ER and how this biology changes in response to changes in cellular environment.
